# Supplementary material for: Diagnosing Progression in Glioblastoma—Tackling a Neuro-Oncology Problem Using Artificial-Intelligence-Derived Volumetric Change over Time on Magnetic Resonance Imaging to Examine Progression-Free Survival in Glioblastoma
Source: Diagnostics (Basel). 2024 Jun 28;14(13):1374. doi: 10.3390/diagnostics14131374 (PMC11241823; doi:10.3390/diagnostics14131374)
Supplement: Supplementary file 1 [file diagnostics-14-01374-s001.zip › diagnostics-3022368-supplementary.pdf]

Supplemental Table S1. MRI Scanning Characteristics

Parameters	T1-pre	T1-post	T2	FLAIR
Acquisition Plane	Axial	Axial	Axial	Axial or Sagittal
Slice Thickness	5mm	5mm	5mm	2mm
In-Plane Resolution	0.625x0.625	0.6875x0.6875	0.4297x0.4297	0.9375x0.9375
Acquisition Matrix	256x259	256x259	384x288	240x240

Supplementary Table S2. Clinical features univariate cox-proportional hazard (cox-PH) ratios for progression-free survival (PFS). HR = odds ratio, SE = standard error. Bold numbers indicate statistically significant values. VMAT (Volumetric Arc Technique), IMRT (Intensity Modulated Radiation Therapy), 3D (3D conformal technique), GTV T1 (Gross Tumor Volume on T1 Gadolinium-enhanced MRI sequence), GTV T2 (Gross Tumor Volume on T2 FLAIR signal sequence), GTR (Gross Total Resection), STR (Subtotal Resection), BX (biopsy).

Variable	Category	6-month PFS			12-month PFS			24-month PFS			Any PFS		
		HR	HR SE	*p value*	HR	HR SE	*p value*	HR	HR SE	*p value*	HR	HR SE	*p value*
**Age**													
		1.028	0.015	0.068	1.021	0.011	**0.049**	1.024	0.009	**0.011**	1.025	0.009	**0.005**
**Gender**													
	Male	reference			reference			reference			reference		
	Female	0.568	0.461	0.219	0.784	0.295	0.409	0.777	0.249	0.311	0.757	0.236	0.238
**Location**													
	Frontal	reference			reference			reference			reference		
	Frontoparietal	0.000	4995.338	0.997	0.722	1.041	0.754	0.433	1.030	0.416	0.662	0.744	0.579
	Frontotemporal	0.273	1.061	0.221	0.475	0.646	0.250	0.792	0.449	0.604	0.649	0.424	0.308
	Occipital	2.042	1.061	0.501	2.908	0.768	0.165	2.984	0.756	0.148	2.700	0.751	0.186
	Occipitoparietal	2.424	0.615	0.150	3.531	0.509	**0.013**	3.609	0.491	**0.009**	3.267	0.483	**0.014**
	Parietal	0.427	0.677	0.208	1.077	0.418	0.859	0.976	0.361	0.946	0.880	0.338	0.704
	Posterior fossa	4.294	1.076	0.176	6.070	1.066	0.091	6.162	1.057	0.085	5.580	1.054	0.103
	Temporal	0.484	0.540	0.179	0.792	0.382	0.543	0.882	0.317	0.693	0.770	0.301	0.384
	Temporoparietal	1.842	0.613	0.320	1.619	0.533	0.366	1.286	0.476	0.597	1.009	0.468	0.984
**Region**													
	Cortical	reference			reference			reference			reference		
	Periventricular	3.348	0.380	**0.001**	2.198	0.274	**0.004**	1.767	0.244	**0.020**	1.438	0.239	0.128
**Resection Status**													
	GTR	reference			reference			reference			reference		
	STR	1.184	0.433	0.697	0.970	0.285	0.915	1.012	0.244	0.960	1.149	0.236	0.555
	Bx	2.662	0.613	0.110	1.307	0.500	0.593	1.044	0.452	0.924	1.140	0.404	0.746
**MGMT methylation status**													
	Methylated	reference			reference			reference			reference		
	Unknown	1.346	0.539	0.581	1.485	0.362	0.275	1.612	0.307	0.120	1.499	0.283	0.153
	Unmethylated	2.248	0.533	0.128	1.949	0.373	0.073	2.354	0.316	**0.007**	2.122	0.298	**0.012**
**Radiation therapy volumes**													
**GTV T2**	10-50 cc	reference			reference			reference			reference		
	50-100 cc	0.857	0.606	0.799	1.752	0.388	0.148	1.300	0.327	0.422	1.115	0.308	0.725
	>100 cc	1.644	0.494	0.314	1.554	0.376	0.241	1.264	0.300	0.435	1.258	0.280	0.414
**GTV T1**	<20 cc	reference			reference			reference			reference		
	20-40 cc	1.519	0.612	0.495	1.599	0.360	0.192	1.384	0.303	0.283	1.374	0.281	0.258
	>40 cc	3.122	0.563	**0.043**	1.812	0.363	0.102	1.766	0.304	0.061	1.769	0.291	**0.050**
**Radiation therapy technique**													
	VMAT	reference			reference			reference			reference		
	IMRT	1.017	0.570	0.977	0.971	0.383	0.940	0.901	0.305	0.731	0.972	0.291	0.923
	3D	2.027	0.521	0.176	2.019	0.358	0.050	1.612	0.298	0.110	1.471	0.292	0.187
**Valproic Acid Administration**													
	No	reference			reference			reference			reference		
	Yes	0.329	0.494	**0.024**	0.581	0.290	0.062	0.606	0.244	**0.040**	0.585	0.232	**0.021**

Supplementary Table S3. Univariable cox-proportional hazard (cox-PH) ratios for AI volumetric estimates and association to progression-free survival (PFS). Δ = change/slope, CET = contrast-enhancing tumor, NET = non-contrast-enhancing tumor, TT = (CET + NET) = total tumor, TB = (Edema + CET + NET) = total burden, HR = odds ratio, SE = standard error. Bold numbers indicate statistically significant values.

Variable			6-month PFS			12-month PFS			24-month PFS			Any PFS		
			HR	HR SE	p-value	HR	HR SE	p-value	HR	HR SE	p-value	HR	HR SE	p-value
**Volumes pre chemoirradiation***														
	**Edema**		0.74858	2.95E-01	0.32695	0.99068	0.17133	0.95642	1.034	0.14598	0.81885	1.04669	0.13939	0.74338
	**CET**		1.28494	2.18E-01	0.25109	1.28622	0.1487	0.0905	1.28704	0.12935	0.05107	1.25459	0.12945	0.07977
	**NET**		1.03962	2.41E-01	0.87168	1.09722	0.14938	0.53453	1.19116	0.12583	0.16446	1.21473	0.12297	0.11366
	**TT**		1.21757	2.19E-01	0.36815	1.24558	0.14595	0.13242	1.29919	0.12679	**0.03898**	1.28723	0.12606	**0.04519**
	**TB**		0.94366	0.25934	0.82306	1.12144	0.16022	0.4744	1.16853	0.13479	0.2479	1.16816	0.13147	0.2371
**Volumes on final MR within analysis interval**														
	**Edema**		1.40848	0.1344	**0.01082**	1.3915	0.11483	**0.00401**	1.30287	0.1135	**0.01975**	1.27066	0.10959	**0.02884**
	**CET**		1.58927	0.14623	**0.00153**	1.4645	0.10528	**0.00029**	1.37035	0.09865	**0.00141**	1.35109	0.09957	**0.00251**
	**NET**		1.43911	0.14225	**0.0105**	1.36031	0.10632	**0.0038**	1.19589	0.09965	0.07264	1.21227	0.09862	0.05095
	**TT**		1.66588	0.15664	**0.00112**	1.46686	0.10752	**0.00037**	1.33913	0.10037	**0.00362**	1.33481	0.1008	**0.00417**
	**TB**		1.53466	0.14169	**0.0025**	1.46415	0.11361	**0.00079**	1.36952	0.11119	**0.00468**	1.33525	0.10828	**0.00758**
**Δ Continuous**														
	**Edema**		1.24254	0.11685	0.06312	1.30384	0.09818	**0.00689**	1.30813	0.09701	**0.00563**	1.31578	0.09529	**0.00398**
	**CET**		0.95238	0.24437	0.84174	1.15303	0.2247	0.52627	1.10568	0.2289	0.66073	1.0601	0.23054	0.80015
	**NET**		1.3163	0.12453	**0.02732**	1.32473	0.11521	**0.01466**	1.31691	0.11445	**0.01616**	1.29001	0.11954	**0.03316**
	**TT**		1.1773	0.23131	0.48041	1.3234	0.18754	0.13514	1.2881	0.19278	0.18909	1.2316	0.19914	0.29552
	**TB**		1.26966	0.12602	0.05815	1.34612	0.10484	**0.00458**	1.34869	0.10408	**0.00405**	1.35377	0.10303	**0.00328**
**Δ Quartiles**														
	**ΔEdema**	**Q1**	Reference			Reference			Reference			Reference		
		**Q2**	0.2456	0.80194	0.07998	1.21356	0.47473	0.68348	0.85298	0.34904	0.64868	1.48125	0.32367	0.22481
		**Q3**	0.73558	0.58577	0.6001	2.94506	0.42889	**0.01179**	2.64258	0.33788	**4.03E-03**	5.14428	0.37068	**9.94E-06**
		**Q4**	2.38571	0.46557	0.06182	5.55626	0.42757	**6.05E-05**	4.45241	0.34844	**1.82E-05**	7.18473	0.37563	**1.52E-07**
	**ΔCET**	**Q1**	Reference			Reference			Reference			Reference		
		**Q2**	0.0731	1.04488	**0.0123**	0.16925	0.51372	**0.00054**	0.1962	0.3702	**1.09E-05**	0.16377	0.36523	**7.28E-07**
		**Q3**	0.15852	0.76921	**0.01664**	0.59361	0.36702	**1.55E-01**	1.05094	0.32434	**8.78E-01**	0.77139	0.30524	**3.95E-01**
		**Q4**	1.49008	0.4047	0.32438	2.15833	0.33684	**0.02237**	2.29264	0.32946	**0.01179**	1.93728	0.31451	**0.0355**
	**ΔNET**	**Q1**	Reference			Reference			Reference			Reference		
		**Q2**	0.16948	0.77514	**0.02203**	0.202	0.51797	**0.00202**	0.1827	0.36784	**3.81E-06**	0.18179	0.36322	**2.68E-06**
		**Q3**	0.26664	0.6588	0.0448	0.84473	0.3555	0.63504	0.5933	0.30736	**8.94E-02**	0.76497	0.3005	**3.73E-01**
		**Q4**	1.50168	0.42086	0.334	1.52581	0.34302	0.21803	1.15912	0.29792	0.62015	1.19821	0.30532	0.55368
	**ΔTT**	**Q1**	Reference			Reference			Reference			Reference		
		**Q2**	0.32403	0.67729	0.09614	0.29583	0.48863	**0.01268**	0.2808	0.36752	**5.48E-04**	0.21242	0.37119	**3.00E-05**
		**Q3**	0.35817	0.67717	0.12946	0.99796	0.36676	9.96E-01	1.37753	0.32154	**3.19E-01**	1.21835	0.3119	**5.27E-01**
		**Q4**	2.31536	0.44497	0.05919	2.44467	0.35023	**0.0107**	2.23296	0.31876	**0.01173**	1.9056	0.31299	**0.03939**
	**ΔTB**	**Q1**	Reference			Reference			Reference			Reference		
		**Q2**	0.23802	0.80196	0.07348	0.53506	0.49304	0.20465	0.71456	0.35529	**3.44E-01**	0.88524	0.31179	**6.96E-01**
		**Q3**	0.58452	0.62698	0.39175	2.24259	0.39857	**4.27E-02**	2.8829	0.3411	**1.91E-03**	2.98905	0.34033	**1.29E-03**
		**Q4**	2.70441	0.46124	**0.03101**	4.84632	0.39805	**7.34E-05**	4.80197	0.35721	**1.12E-05**	5.15277	0.3532	**3.45E-06**

Supplemental Table S4. Multivariable cox-proportional hazard (cox-PH) ratios for AI volumetric estimates and association to progression-free survival (PFS), adjusted for clinical features of known important (Age, MGMT status, tumor region, VPA usage, and GTV T1 volume). Δ = change/slope, CET = contrast-enhancing tumor, NET = non-contrast-enhancing tumor, TT = (CET + NET) = total tumor, TB = (Edema + CET + NET) = total burden, HR = hazard ratio, SE = standard error. Bold numbers indicate statistically significant values.

Variable			6-month PFS			24-month PFS		
			Adj HR	SE	p-value	Adj HR	SE	p-value
**Volumes on final MR within analysis interval**								
	**Edema**					0.3856	0.2156	**0.01975**
**Δ Continuous**								
	**Edema**					1.9757	0.1957	**0.0005**
**Δ Quartiles**								
	**ΔCET**	**Q1**		Reference			Reference	
		**Q2**	0.08364	1.0716	**0.0206**	0.0756	0.4998	**2.39E-07**
		**Q3**	0.02212	0.9046	0.0955	2.2129	0.4728	0.093
		**Q4**	1.3055	0.5356	0.6187	3.7673	0.4857	**0.006**
	**ΔNET**	**Q1**					Reference	
		**Q2**				0.0951	0.5008	**2.63E-06**
		**Q3**				0.197	0.3929	**3.55E-05**
		**Q4**				1.3497	0.4038	0.4577

Supplemental Table S5. Spearman rank correlation coefficient for association between clinical features and AI-based volume metrics. Δ = change/slope, CET = contrast-enhancing tumor, NET = non-contrast-enhancing tumor, TT = (CET + NET) = total tumor, TB = (Edema + CET + NET) = total burden

Feature	6 months					All time				
	**ΔCE**	**ΔNET**	**ΔED**	**ΔTT**	**ΔTB**	**ΔCE**	**ΔNET**	**ΔED**	**ΔTT**	**ΔTB**
Age	-0.06	-0.006	0.185	-0.093	0.145	-0.127	-0.084	0.239	-0.182	0.132
GTV T1	-0.175	-0.179	0.3	-0.19	0.186	-0.136	-0.186	0.172	-0.126	0.095

Supplemental Table S6. Wilcoxon rank-sum test for association between clinical groups and AI-based volume metrics. All reported values reflect p-value from statistical test. Δ = change/slope, CET = contrast-enhancing tumor, NET = non-contrast-enhancing tumor, TT = (CET + NET) = total tumor, TB = (Edema + CET + NET) = total burden

Feature	6 months					All time				
	**ΔCE**	**ΔNET**	**ΔED**	**ΔTT**	**ΔTB**	**ΔCE**	**ΔNET**	**ΔED**	**ΔTT**	**ΔTB**
MGMT	0.3496	0.2345	0.3722	0.6395	0.3527	0.0342	0.5817	0.1087	0.09	0.077
VPA	0.5635	0.9238	0.693	0.3947	0.6386	0.2411	0.7447	0.8835	0.1566	0.5688

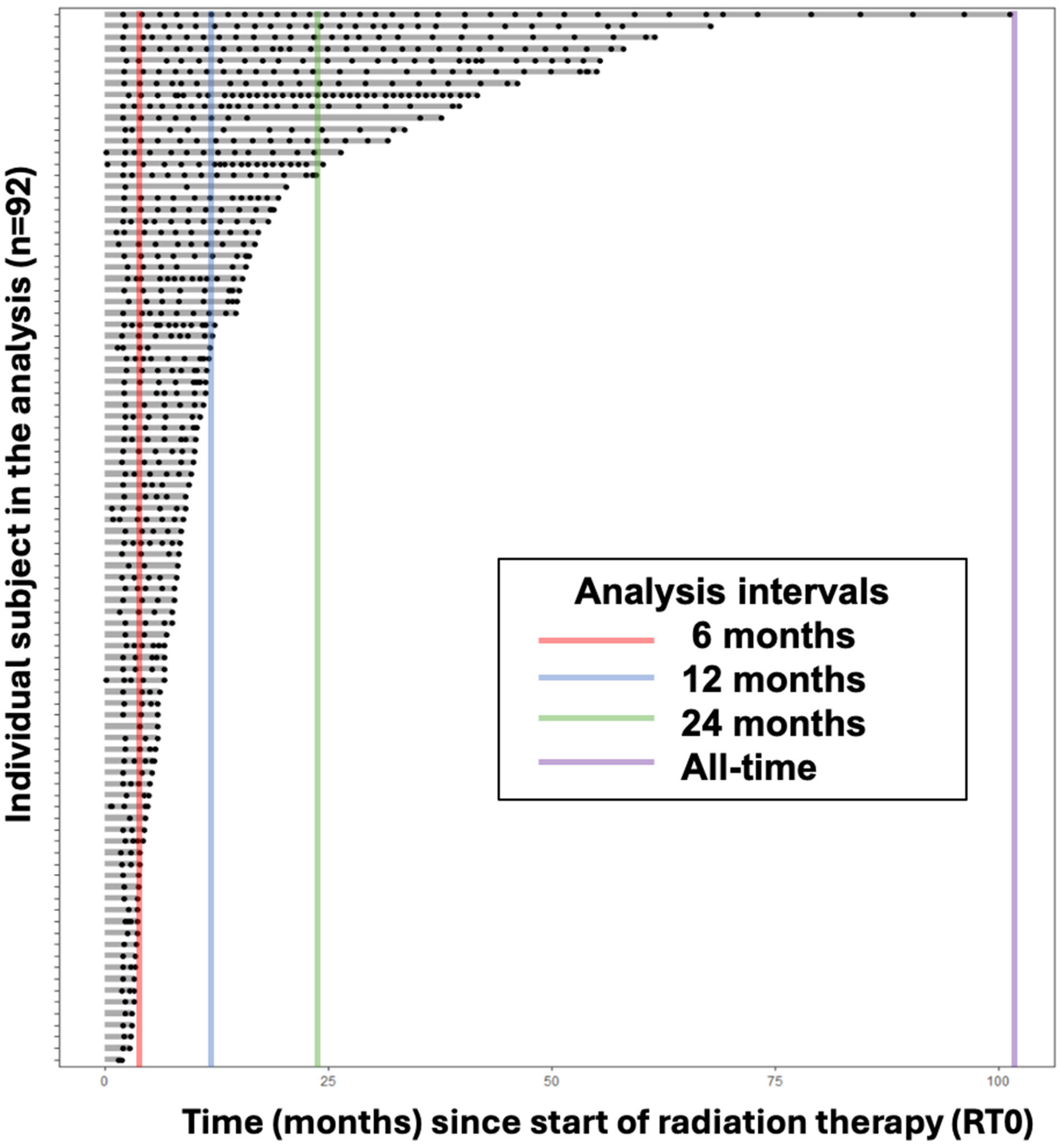

Supplemental Figure S1. Follow-up scan distribution for the included patients, within analysis intervals highlighted by vertical lines. For a given analysis interval, any scan between RT0 (start of RT) and interval endpoint (+/- 1.5 months) are utilized.

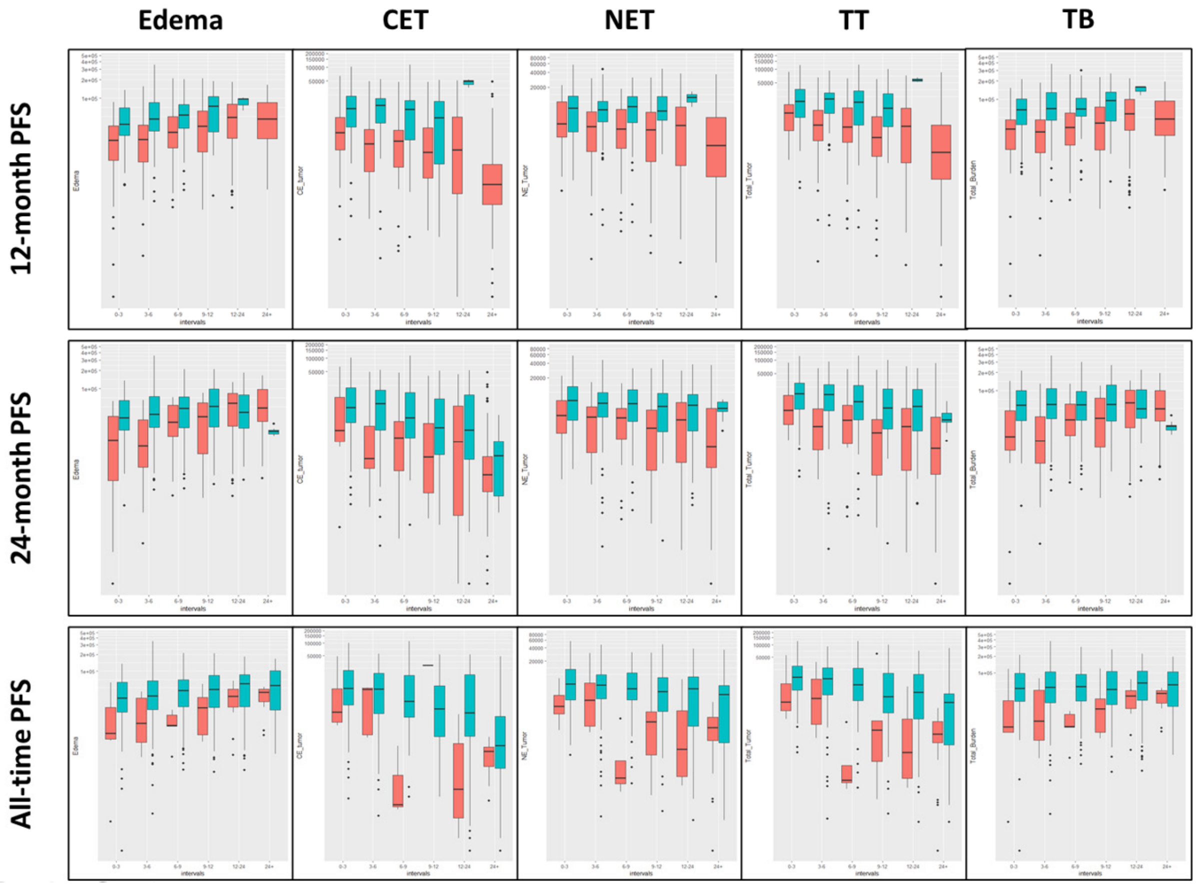

Supplemental Figure S2. AI volume components plotted for each progressive-free-survival time interval. Volume (y-axis) is in units of mm3 and time (x-axis) is in units on months. The y-axis was transformed with the log function. CET = contrast-enhancing tumor, NET = non-contrast enhancing tumor, TT = (CET+NET) = total tumor volume, TB = (Edema + CET + NET) = total volume burden, PFS = progression-free survival, PFS 0 = non-progressors (orange-red), PFS 1 = progressors (blue-cyan).

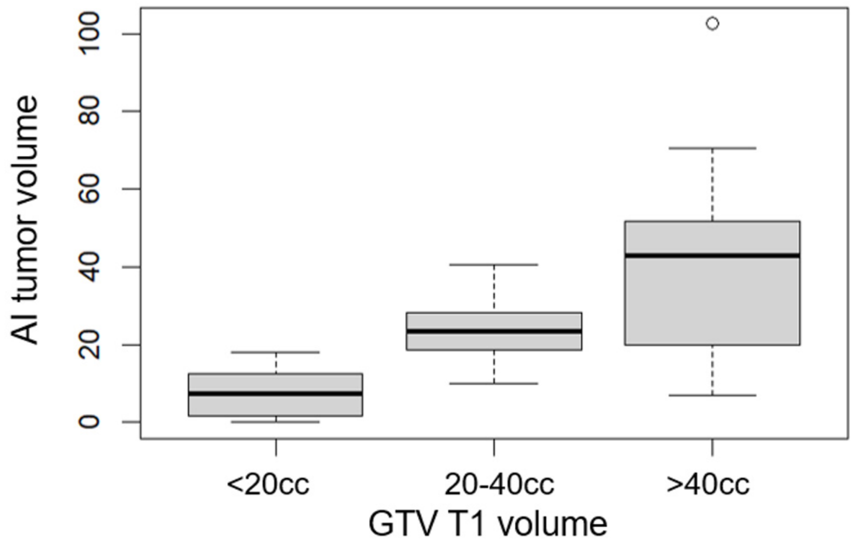

Supplemental Figure S3. Association of AI total tumor volume (CET+NET) prior to chemo-irradiation with GTV T1 volume delineated by physician for treatment planning.

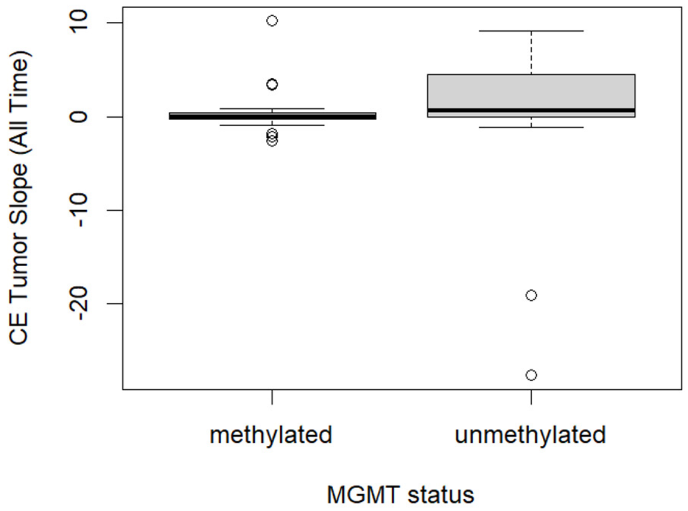

Supplemental Figure S4. Association of ΔCET tumor volume from all-time analysis with patient MGMT status, demonstrating a significant association by Wilcoxon rank sum test.

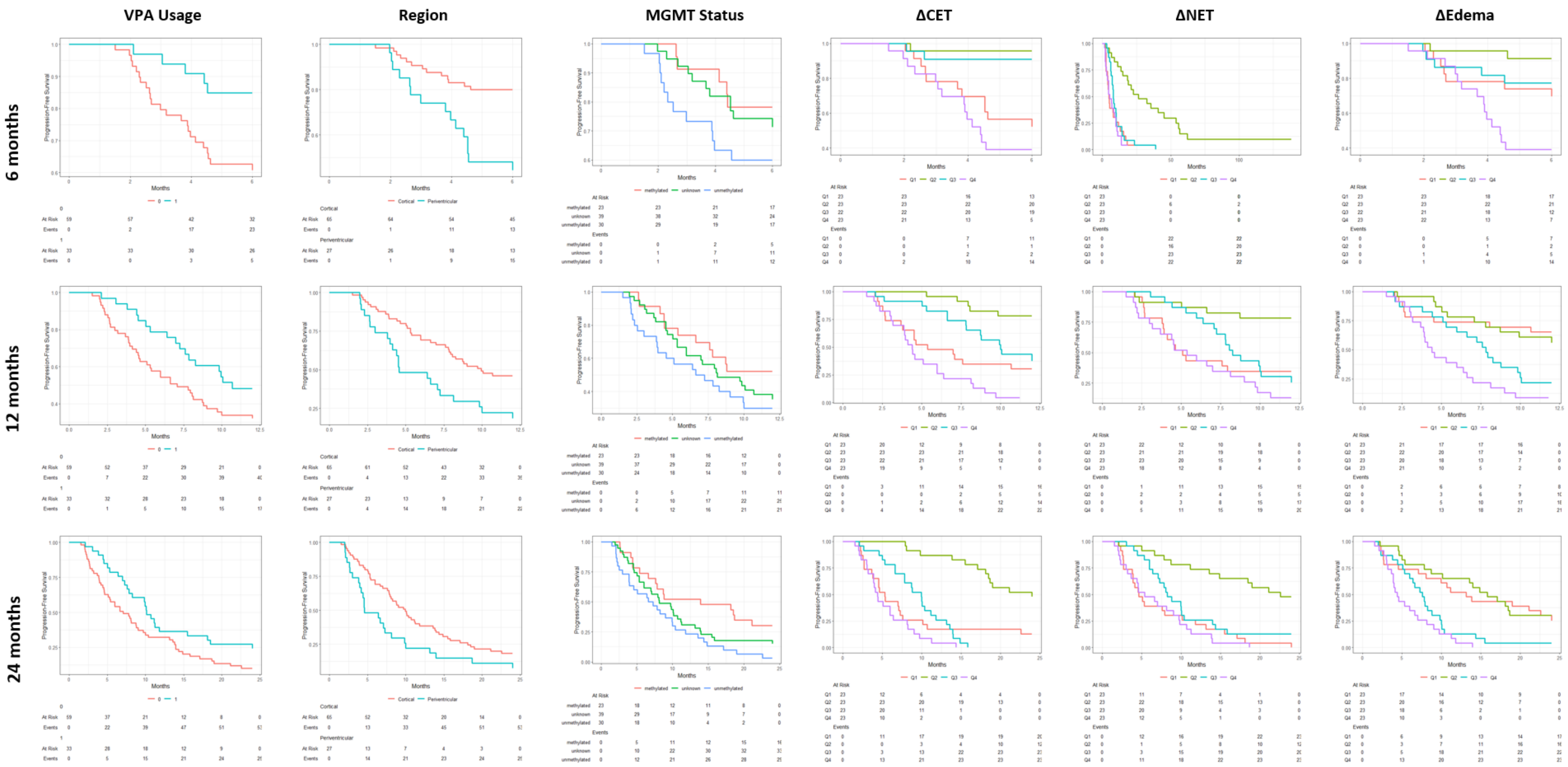

Supplemental Figure S5. Kaplan-Meier plots for all interval analysis progression free survival prediction. ***From left to right:*** KM plots clinical variables: VPA, Tumor Region, and MGMT status, followed by AI volumetric slopes: ΔCET, ΔNET, and ΔEdema. ***From top to bottom:*** time intervals considered: 6-months, 12-months, and 24-months. VPA = valproic acid administration, CET = contrast-enhancing tumor, NET = non-contrast enhancing tumor, cat = categorical, PFS = progression-free survival
